# New Books

**Published:** 2011-05

**Climate Change and Society**

John Urry

Hoboken, NJ:John Wiley & Sons, 2011. 200 pp. ISBN: 978-0-7456-5036-4, $69.95

**Comprehensive Toxicology, 2nd ed.**

Charlene A. McQueen

St. Louis, MO:Elsevier, 2010. 250 pp. ISBN: 978-0-08-046868-6, $6495

**Corruption, Development and the Environment**

Lorenzo Pellegrini

New York:Springer, 2011. 195 pp. ISBN: 978-94-007-0598-2, $129

**Ecotoxicology**

Erik Jorgensen, ed.

St. Louis, MO:Elsevier, 2010. 402 pp. ISBN: 978-0-444-53628-0, $79.95

**Energy Conservation in East Asia: Towards Greater Energy Security**

Elspeth Thomson, Youngho Chang, Jae-Seung Lee, eds.

Hackensack, NJ:World Scientific, 2010. 408 pp. ISBN: 978-981-277-177-3, $135

**Energy, Sustainability and the Environment: Technology, Incentives, Behavior**

Fereidoon Perry Sioshansi, ed.

St. Louis, MO:Elsevier, 2011. 640 pp. ISBN: 978-0-12-385136-9, $99.95

**Environanotechnology**

Maohong Fan, C.P. Huang, Alan E. Bland, Zhonglin Wang, Rachid Slimane, Ian G. Wright, eds.

St. Louis, MO:Elsevier, 2010. 310 pp. ISBN: 978-0-08-054820-3, $147

**Fundamentals of Ecosystem Science**

Kathie C. Weathers, David L. Strayer, Gene E. Likens, eds.

St. Louis, MO:Elsevier, 2011. 450 pp. ISBN: 978-0-12-088774-3, $64.95

**Greening of Petroleum Operations: The Science of Sustainable Energy Production**

M.R. Islam, A.B. Chhetri, M.M. Khan

Hoboken, NJ:John Wiley & Sons, 2010. 859 pp. ISBN: 978-0-470-62590-3, $239

**Managing CO_2_** **Emissions in the Chemical Industry**

Hans-Joachim Leimkühler, ed.

Hoboken, NJ:John Wiley & Sons, 2010. 480 pp. ISBN: 978-3-527-32659-4, $195

**Oil Spill Science and Technology**

Mervin Fingas, ed.

St. Louis, MO:Elsevier, 2011. 1,192 pp. ISBN: 978-1-85617-943-0, $200

**Perspectives from United Kingdom and United States Policy Makers on Obesity Prevention**

Paula Tarnapol Whitacre and Annina Catherine Burns, eds.

Washington, DC:National Academies Press, 2010. 96 pp. ISBN: 978-0-309-15078-1, $21

**Reproductive and Developmental Toxicology**

Ramesh C. Gupta, ed.

St. Louis, MO:Elsevier, 2011. 1,220 pp. ISBN: 978-0-12-382032-7, $250

**Risks of Hazardous Wastes**

Paul E. Rosenfeld, Lydia Feng

St. Louis, MO:Elsevier, 2011. 472 pp. ISBN: 978-1-4377-7842-7, $195

**Towards a Sustainable Asia: Energy**

Association of Academies of Sciences in Asia

New York:Springer, 2011. 56 pp. ISBN: 978-3-642-16680-8, $139

**The Challenges of Climate Change: Which Way Now?**

Daniel P. Perlmutter, Robert L. Rothstein

Hoboken, NJ:John Wiley & Sons, 2011. 248 pp. ISBN: 978-0-470-65497-2, $39.95

**The Chemical Element: Chemistry’s Contribution to Our Global Future**

Javier Garcia-Martinez, Elena Serrano-Torregrosa, eds.

Hoboken, NJ:John Wiley & Sons, 2011. 368 pp. ISBN: 978-3-527-32880-2, $34.50

